# Evaluator effect on the ultrasound measurement of subcutaneous fat deposition and loin eye area from weaning to slaughter lambs

**DOI:** 10.14202/vetworld.2021.259-264

**Published:** 2021-01-28

**Authors:** Fernando Miranda de Vargas Junior, Tatiane Fernandes, Alexsander Toniazzo de Matos, Alexandre Rodrigo Mendes Fernandes, Luis Gustavo Castro Alves, Julianna Andrade Rossatti, Gustavo Daniel Vega Britez, Natássia Gabriela Targanski Zagonel

**Affiliations:** 1 Post-graduate Program in Animal Science, Federal University of Grande Dourados, Dourados, MS, 79825-070, Brazil; 2 University Center of Grande Dourados - UNIGRAN, Dourados, MS, 79824-900, Brazil; 3 Facultad de Ciencias Agrarias, Universidad Nacional de Asunción Filial Pedro Juan Caballero, Pedro Juan Caballero, 79900-000, Paraguay

**Keywords:** accuracy, identity test, precision, repeatability, reproducibility

## Abstract

**Background and Aim:**

Ultrasound is a non-invasive technique that enables animal evaluation and body condition classification of animals. Although it is not difficult to obtain an image, the analysis of this image can influence results quality. This study aimed to evaluate the repeatability and reproducibility of the technician trained in image interpretation obtained using different ultrasound frequencies.

**Materials and Methods:**

Ninety-six lambs were used, ranging in weight from 15 to 40 kg. The images were captured using a linear probe of 13 cm, with a 3.5 megahertz (MHz) frequency and an acoustic couplant aid “standoff” or using a multifrequency transducer (6 and 8 MHz), on B mode, with a linear probe and 8.0 MHz frequency. All measurements were performed by the same technician, on the left side, between the 12^th^ and 13^th^ rib. Five different evaluators, at two different times, with aid of Image J software measured the loin eye area (LEA; only for images obtained with 3.5 MHz), *Longissimus thoracis et lumborum* depth (DLM), subcutaneous fat thickness (SFT), subcutaneous fat thickness plus skin (SFST), and skin thickness (ST).

**Results:**

For LEA, DLM, SFT, SFST, and ST, variation was observed (p<0.01) between evaluators; however, there was no difference (p>0.05) between the 2 times of evaluation. Images measurements obtained with a frequency of 8.0 MHz had better repeatability indices and reproducibility indices. Accordingly, the identity test demonstrated that measurements performed on images obtained using 3.5 or 8.0 MHz were not equivalent.

**Conclusion:**

Ultrasound image measurements obtained using an 8.0 MHz frequency were more accurate and precise. It is important to use only one evaluator or providing the simultaneous training for all evaluators.

## Introduction

Ultrasound is a non-invasive technique that enables animal evaluation and body condition classification of animals into those for slaughter and those for reproduction. Livestock production systems have started to assess subcutaneous fat thickness (SFT) using ultrasound imaging to predict carcass tissue composition of animals *in vivo* and to indicate slaughter time [1]. Ultrasound also helps in the breeding stock selection and can indicate precocity and earning potential of weights, feed efficiency, and income from contemporary animal cuts. Sheep from different genetic groups can be classified as early, intermediate, or late, depending on the SFT deposited as the animal matures [2]. Furthermore, it can measure the energy reserves on reproduction stages; ultrasound measurements allow the producer to make the appropriate decisions for proper management to conditioning animals according to their physiological stage.

According to McManus *et al*. [3], the SFT measured between 12^th^ and 13^th^ ribs has a high and positive correlation with carcass fat. Loin eye area (LEA) measure indicates the amount of marketable meat, and the *Longissimus thoracis et lumborum* (DLM) depth can predict the amount of muscle in the carcass [4,5]. Lambs that had a higher LEA were more efficient and showed a better performance in confinement, resulting in heavier castings [6]. The skin thickness (ST) could be used in the equation to estimate warm and cold carcass weights [7]. These variables can be measured using ultrasound. Therefore, precise measurement of these characteristics is crucial for production estimate accuracy, as well as for decision-making regarding the choice between reproduction and slaughter. An ultrasound image can be obtained by different frequencies ranging from 3.5 to 10 megahertz (MHz), which allows for greater accuracy and precision when examining the target anatomical region. Frequencies higher than 5 MHz generate high-resolution images but have a lower penetration, and therefore do not allow LEA visualization [8]. Frequencies lower than 5 MHz allow a deeper view but generate worse quality images [9]. Although it is not difficult to obtain an image, the analysis of this image also seems to influence results quality [10].

For cattle, it has been reported that the structures that divide the tissues often have variable dimensions with different acoustic impedances, which can result in differences between operators when interpreting the images [11]. According to Silva [8], anatomy knowledge; prior involvement in carcass work (especially dissection); and familiarity with the equipment, image acquisition, and interpretation are some of the factors that pose potential problems related to the operator. Thus, this study aimed to evaluate the influence of image capture frequency (MHz) on the repeatability and reproducibility of the technician trained (image evaluator) in the interpretation of lamb ultrasound images.

## Materials and Methods

### Ethical approval

Experimental protocols were approved by the Committee of Ethics in Animal Experimentation (CEUA; protocol no. 018/2013) of the Federal University of Grande Dourados (UFGD), Dourados, Mato Grosso do Sul, Brazil.

### Study period and location

The experiment was carried out in September 2013, at the Animal Science sector of the Faculty of Agricultural Sciences of the Federal University of Grande Dourados - FCA / UFGD, located in the municipality of Dourados, Mato Grosso do Sul, Brazil (22°11′55”S, 54°56′7”W and 452 m altitude).

### Animals and Images capture

We used 96 male uncastrated lambs of the Pantaneira breed, with weight varying from 15 to 40 kg. As treatments, 3.5 or 8.0 MHz frequencies were used to collect ultrasound images. Images were captured using two types of ultrasound equipment: One of the brand Aloka (SSD-500v Aloka Co., Ltd, Mitaka-shi, Tokyo, Japan), with a linear probe of 13 cm, with 3.5 MHz frequency and the support of acoustic coupling “standoff,” and another of the brand Pie Medical (410477 Falco 100 rev A, California Prop 65 Warning, US) with a multifrequency transducer (6 and 8 MHz), using B mode, with a linear probe and 8.0 MHz frequency. To perform the measurements, lambs were manually immobilized, and with the aid of a comb, the wool was separated in the measuring areas and mucilage was applied for the best transducer coupling to the skin [12]. All the measurements were performed by the same technician, on the left side, between 12^th^ and 13^th^ ribs, 4 cm from the spine median line. Images generated by ultrasound were digitally stored for further analysis using a video capture card [13].

### Image evaluation

Images were analyzed by five different evaluators at 2 different times. The five evaluators were trained to use Image J software (National Institute of Mental Health, Bethesda, Maryland, USA - http://rsb.info.nih.gov/nih-image/), all evaluators had experience in evaluating ultrasound images from other experiments, but no simultaneous training was performed with the evaluators before starting these evaluations.

**Figure-1:**
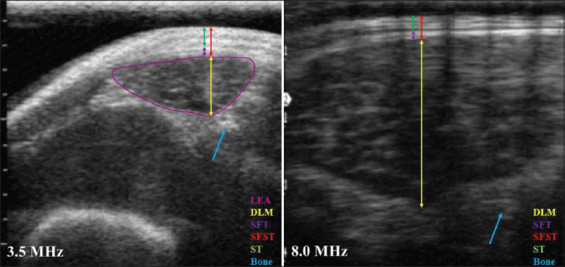
Ultrasound image obtained with 3.5 or 8.0 MHz frequency. Indicating measurements of loin eye area, *Longissimus thoracis et lumborum* muscle depth, subcutaneous fat thickness, subcutaneous fat plus skin thickness, skin thickness.

Image J software was used for evaluation of the ultrasound images by each evaluator. For all images, a scale adjustment of 30 pixels/cm was performed. Measurements of LEA (only for images obtained with 3.5 MHz), DLM, SFT, SFT plus ST (SFST), and ST were performed. LEA was determined by muscle area contour on the images, DLM was obtained by measuring muscle thickness between the fat layer and the muscle end, SFT was obtained by measuring adipose tissue that was between *Longissimus thoracis et lumborum* muscle and skin, and SFST was obtained by measuring SFT plus ST (Figure-1).

### Statistical analysis

Data were evaluated using the Minitab program 17.x. Measurement repeatability and reproducibility taken by five different evaluators was determined, considering measurement system as: Acceptable (<1% variation in the process), ponderable, use is conditioned to the ultrasound evaluation applicability (between 1 and 9 % variation in the process), or unacceptable (more than 9 % variation in the process) [14]. To compare the results between frequencies (3.5 or 8.0 MHz) used to collect the images, Pearson’s correlation was calculated in addition to identity test proposed by Leite and Oliveira [15] using Mann–Whitney and Wilcoxon statistical tests.

## Results

For the parameters of LEA, DLM, SFT, SFST, and ST obtained with 3.5 MHz frequency, a variation (p<0.01) was observed among evaluators, with no difference (p>0.05) between assessments performed at different times by the same evaluator (Table-1). Frequency of 8.0 MHz makes it possible to obtain a sharper image (Figure-1) but with a lower depth range.

**Table-1:** Amplitude and average values and evaluation of the repeatability and reproducibility obtained by different *evaluators to loin eye area, Longissimus thoracis et lumborum* muscle depth, subcutaneous fat thickness, subcutaneous fat plus skin thickness, skin thickness, based images generated with 3.5 MHz frequency.

	Evaluator					Mean	SEM	p-value	Evaluator	Standard Variation (%)	
		
	1	2	3	4	5			Repetition		Repeatability	Reproducibility
Loin eye area (cm^2^)										
Minimum	3.40	3.02	3.00	2.03	1.97	1.97	2.336	0.96	<0.01	13.30	4.99
Mean	7.41	8.53	8.87	7.67	6.77	7.85					
Maximum	12.16	12.98	14.74	14.90	10.61	14.90					
*Longissimus thoracis et lumborum* muscle depth (cm)										
Minimum	1.17	1.00	0.87	1.10	0.69	0.69	0.373	0.64	<0.01	2.12	0.82
Mean	2.03	2.09	1.90	1.92	1.72	1.93					
Maximum	2.80	2.70	2.60	2.65	2.44	2.80					
Subcutaneous fat thickness (mm)										
Minimum	0.13	0.23	0.11	0.20	0.03	0.03	0.204	0.21	<0.01	0.69	1,13
Mean	0.28	0.51	0.26	0.61	0.15	0.36					
Maximum	0.80	0.83	0.52	1.17	0.39	1.17					
Subcutaneous fat plus skin thickness (mm)										
Minimum	0.40	0.43	0.23	0.47	0.09	0.09	0.264	0.55	<0.01	0.92	1.45
Mean	0.75	0.82	0.48	0.84	0.29	0.64					
Maximum	1.70	1.23	0.87	1.54	1.00	1.70					
Skin thickness (mm)										
Minimum	0.06	0.07	0.01	0.01	0.01	0.01	0.155	0.84	<0.01	0.64	0.75
Mean	0.47	0.30	0.22	0.24	0.14	0.27					
Maximum	0.90	0.53	0.52	0.78	0.71	0.90					

SEM=Standard error mean

When 8.0 MHz frequency was used for image collection, measurements of DLM, SFT, SFST, and ST showed a difference (p<0.01) between evaluators, with no difference (p≥0.05) between assessments performed at different times by the same evaluator (Table-2), similar to the results obtained from images collected using a 3.5 MHz frequency.

**Table-2:** Amplitude and average values and evaluation of the repeatability and reproducibility obtained by different evaluators to loin eye area, *Longissimus thoracis et lumborum* muscle depth, subcutaneous fat thickness, subcutaneous fat plus skin thickness, skin thickness, based images generated with 8.0 MHz frequency.

	Evaluator					Mean	SEM	p-value	Evaluator	Standard variation (%)	
		
	1	2	3	4	5			Repetition		Repeatability	Reproducibility
*Longissimus thoracis et lumborum* muscle depth (cm)										
Minimum	1.33	1.24	1.22	1.33	0.42	0.42	0.433			2.40	1.10
Mean	2.12	2.13	1.98	2.06	1.68	1.99		0.06	<0.01		
Maximum	2.64	2.79	2.72	2.74	2.74	2.79					
Subcutaneous fat thickness (mm)										
Minimum	0.09	0.06	0.13	0.04	0.01	0.01	0.096			0.46	0.39
Mean	0.23	0.17	0.27	0.19	0.10	0.19		0.42	<0.01		
Maximum	0.55	0.34	0.48	0.61	0.28	0.61					
Subcutaneous fat plus skin thickness (mm)										
Minimum	0.27	0.13	0.22	0.10	0.10	0.10	0.150			0.63	0.71
Mean	0.52	0.29	0.40	0.28	0.22	0.35		0.29	<0.01		
Maximum	0.90	0.50	0.73	0.70	0.86	0.90					
Skin thickness (mm)										
Minimum	0.06	0.03	0.01	0.20	0.03	0.01	0.098			0.41	0.47
Mean	0.29	0.12	0.14	0.09	0.13	0.15		0.47	<0.01		
Maximum	0.49	0.22	0.53	0.22	0.58	0.58					

SEM=Standard error mean

For LEA measurement, repeatability was unacceptable (above 9% variation) and reproducibility was ponderable (between 1 and 9% variation) depending on the application. When repeatability or reproducibility is considered ponderable, it indicates that this evaluation can be used depending on its application, in situations such as scientific research, the use would not be recommended, but in field situations, for lot division, diet adjustment, and breeding season beginning, this evaluation does not need much accuracy, so the tool could be used. For DLM obtained from 3.5 MHz images, repeatability was ponderable depending on the application, and the reproducibility was considered acceptable (<1% variation). For SFT and SFST measurements obtained from 3.5 MHz images, repeatability indices were considered acceptable, and reproducibility indices were ponderable, indicating that measurement system is acceptable depending on the application. For ST evaluation, both repeatability and reproducibility were considered acceptable.

When repeatability and reproducibility test was applied to the measurements taken from images obtained with 8.0 MHz, a higher precision and accuracy of assessments were observed. For DLM, repeatability and reproducibility were ponderable depending on the application. For SFT, SFST, and ST measurements obtained from images using 8.0 MHz, repeatability and reproducibility indices were considered acceptable.

**Figure-2:**
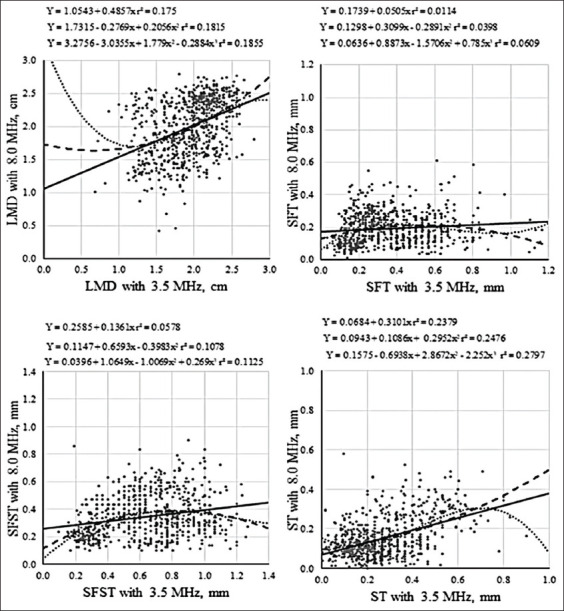
Pearson linear correlation for measurements of *Longissimus thoracis et lumborum* muscle depth, subcutaneous fat thickness, subcutaneous fat plus skin thickness, skin thickness, based images generated with 3.5 or 8.0 MHz frequency.

Correlations between DLM, SFT, SFST, and ST measurements performed on images obtained with 3.5 or 8.0 MHz frequency were low but significant, ranging from 0.11 to 0.49 (Table-3). When DLM, SFT, SFST, and ST data were plotted to generate a linear equation (Figure-2), a low coefficient of determination was observed. Dispersion between maximum and minimum values for LEA, DLM, SFT, SFST, and ST is dependent on the variation in physiological state of evaluated animals, since animals varied in weight from 15 to 40 kg. Since our aim was to evaluate a representative population, we decided to use animals that presented dispersion in measured parameters. The relationship between data obtained using different frequencies (3.5 and 8.0 MHz) does not fit first, second, or third-degree regression equations (Figure-2).

**Table-3:** Pearson correlation, Mann–Whitney and Wilcoxon test of the measurements obtained by different evaluators to LMD, SFT, SFST, and ST based images generated with 3.5 or 8.0 MHz frequency.

Frequency	Pearson correlation	p-value			Conclusion

		Pearson	Mann-Whitney	Wilcoxon	
DLM					
3.5	0.418	<0.01	<0.01	<0.01	3.5≠8.0
8.0					
SFT					
3.5	0.107	0.01	<0.01	<0.01	3.5≠8.0
8.0					
SFST					
3.5	0.240	<0.01	<0.01	<0.01	3.5≠8.0
8.0					
ST					
3.5	0.488	<0.01	<0.01	<0.01	3.5≠8.0
8.0					

n=740 measurements for variable. LMD=*Longissimus thoracis et lumborum* muscle depth, SFT=Subcutaneous fat thickness, SFST=Subcutaneous fat plus skin thickness, ST=Skin thickness

## Discussion

The difference of LEA, DLM, SFT, SFST, and ST indicates that evaluators were consistent in their assessments; however, measurements were not consistent between evaluators, regardless of which variable was measured. According to Mercadante *et al*. [11], implementation of systems for carcass evaluation by ultrasonography is dependent on the availability of a high number of trained technicians, both to collect images in the field and to measure images in the laboratory. The ability to interpret ultrasonographic image depends on the operator’s experience [8]. All evaluators in this study had previous experience in ultrasound images evaluation. However, the training received by them was not simultaneous, which may have caused consistency in the evaluations of the same technician (when they were repeated at different times), but variation in result quality between evaluators.

Differences in measurements between evaluators might be due to anatomical points that are difficult to visualize, for example, in ultrasound image of *Longissimus thoracis et lumborum* muscle, its lateral and inferior borders often have poor resolution [9]. These difficult-to-visualize points will interfere mainly with LEA and DLM measurements, which had the least satisfactory results regarding repeatability and reproducibility.

A low-frequency probe has a low resolution of surface tissue layers, for example, for subcutaneous fat measurement, whereas a high-frequency probe has a higher resolution at the surface and lower penetration capacity [8]. Therefore, it is not possible to evaluate LEA obtained from images using an 8.0 MHz frequency. Furthermore, image quality collected in small ruminants can be affected by narrow space between ribs and also by muscle small area [16] and wool presence of, which needs to be removed at the image collection site [17].

Repeatability and reproducibility test evaluate not only the difference between assessments but also assessment precision and accuracy. Repeatability and reproducibility results indicate that when assessment accuracy and precision are fundamental, for example, in scientific study cases, the use of 8.0 MHz frequency is the most appropriate.

When identity test was performed, the significance of Mann–Whitney and Wilcoxon tests, which compare correlation coefficients and mean errors, was used to determine the similarity or identity between methods. This evaluation demonstrated a significant effect for all comparisons between 3.5 and 8.0 MHz frequencies. Observing the criteria established by Leite and Oliveira [15], we can conclude that measurements performed on images obtained using 3.5 or 8.0 MHz are not equivalent.

In the present study, we can observe that both linear correlation and identity test obtained from different frequencies (3.5 and 8.0 MHz) presented different results, and it is not possible to use equations to predict equivalence between measurements performed on images obtained with 3.5 or 8.0 MHz frequencies. Considering the observed variations, it seems that it is fundamental to scan the images for further evaluation in a quiet environment and with appropriate software, since the greatest evaluation accuracy can be obtained using specific software depending on the image resolution [8]. It helps to explain some of the results, where the ultrasound measurements were unsatisfactory compared to subsequent assessments obtained directly on the carcass [18].

## Conclusion

Ultrasound can be an asset for producers since they can predict the carcass and meat characteristics, facilitating their management. However, it is important to highlight that the ultrasonographic image measurements obtained with a frequency of 8.0 MHz are more accurate and precise than those from images obtained using 3.5 MHz. Depth variables of *Longissimus thoracis et lumborum* muscle, fat thickness, and fat thickness plus skin were more accurate and precise when 8.0 MHz was used. The identity test indicated no equivalence between measurements obtained with 3.5 and 8.0 MHz frequencies. A single evaluator or a set of trained technicians that are constantly evaluated is required for technical standardization and enough training to reduce the evaluation dispersion and to be a reliable work.

## Authors’ Contributions

FMVJ, ATM, ARMF: Conception and design of the study. LGCA, JAR, GDVB, NGTZ: Acquisition of the data. FMVJ, TF, ATM: Analysis and interpretation of the data. TF, ATM, LGCA, JAR, GDVB, NGTZ: Drafted and revised the manuscript. FMVJ, ARMF: Revised critically for important intellectual content All authors read and approved the final manuscript.
